# Abundant box jellyfish, *Chironex* sp. (Cnidaria: Cubozoa: Chirodropidae), discovered at depths of over 50 m on western Australian coastal reefs

**DOI:** 10.1038/srep22290

**Published:** 2016-02-29

**Authors:** John K. Keesing, Joanna Strzelecki, Marcus Stowar, Mary Wakeford, Karen J. Miller, Lisa-Ann Gershwin, Dongyan Liu

**Affiliations:** 1 CSIRO Oceans and Atmosphere, Private Bag 5, Wembley, 6913, Australia; 2 Australian Institute of Marine Science (AIMS), University of Western Australia Oceans Institute, (MO96), 35 Stirling Highway, Crawley, 6009, Australia; 3 CSIRO Oceans and Atmosphere, GPO Box 1538, Hobart, 7001, Australia; 4 Yantai Institute of Coastal Zone Research, Chinese Academy of Sciences, 264003, Yantai, Shandong, P. R. China; 5 The Western Australian Marine Science Institution, Perth, Australia

## Abstract

Box jellyfish cause human fatalities and have a life cycle and habit associated with shallow waters (<5 m) in mangrove creeks, coastal beaches, embayments. In north-western Australia, tow video and epibenthic sled surveys discovered large numbers (64 in a 1500 m tow or 0.05 m^−2^) of *Chironex* sp. very near to the benthos (<50 cm) at depths of 39–56 m. This is the first record of a population of box jellyfish closely associated with the benthos at such depths. *Chironex* were not widespread, occurring only in 2 of 33 tow videos and 3 of 41 epibenthic sleds spread over 2000 km^2^. All *Chironex* filmed or captured were on low to medium relief reefs with rich filter feeder communities. None were on soft sediment habitat despite these habitats comprising 49% of all sites. The importance of the reef habitat to *Chironex* remains unclear. Being associated with filter feeder communities might represent a hazard, and other studies have shown *C. fleckeri* avoid habitats which represent a risk of entanglement of their tentacles. Most of our observations were made during the period of lowest tidal current flow in the morning. This may represent a period favourable for active hunting for prey close to the seabed.

Cubozoans or box jellyfish include members of the highly venomous genus *Chironex* and have been responsible for numerous fatalities in tropical and subtropical coastal regions of the world. The most notorious of these is *Chironex fleckeri*, which has been responsible for 77 deaths in Australia alone^1^,^2^. *C. yamaguchii* has been responsible for fatalities in Japan, and probably the Philippines^3^,^4^. Despite their notoriety, many aspects of the behaviour and ecology of *Chironex* remain a mystery, and this is true for cubozoan jellyfish in general^5^. Most observations of *Chironex* have been made in shallow coastal waters where they are a hazard for swimmers, and where known, their sessile polyp stage occurs in mangrove creeks and the medusa stage enables them to spread among shallow coastal areas^6^,^7^. In comparison, observations of *Chironex* in deeper shelf waters have hitherto been limited to reports of catches by prawn trawlers^7^ without any detail on where in the water column the medusae entered the fishing nets. Extensive surveys^8^ on the Great Barrier Reef did not find any evidence of *C. fleckeri* away from the mainland or large continental islands or in depths >5 m. Earlier 47 offshore surveys over 2 years taking 1200 surface and subsurface plankton samples (4000 cubic meters) captured only 8 *C. fleckeri*^6^. However, other species of box jellyfish have been recorded from deepwater. The carybdeid *Alatina alata* was recorded to 675 m^9^ indicating that cubozoans can occur across a wide range of depths.

The opportunity to observe *Chironex* on deep coastal reefs arose during work for a large scale survey using an underwater towed video camera and epibenthic sled to characterise the sea bed habitats and biodiversity of Camden Sound, a remote, previously unsurveyed location in the Kimberley region of north-western Australia. During the survey of March 2015, one of the routine video stations discovered large numbers of box jellyfish close to the seabed in over 40 m of water. Epi-benthic sleds carried out at stations nearby also caught box jellyfish. This paper is the first to report on large numbers of box jellyfish close to the benthos on deep reefs (40+ m) with rich filter feeder communities, and our objective is to document this phenomenon as a contribution to the scant information on box jellyfish habitat.

## Results

**Figure 1:**
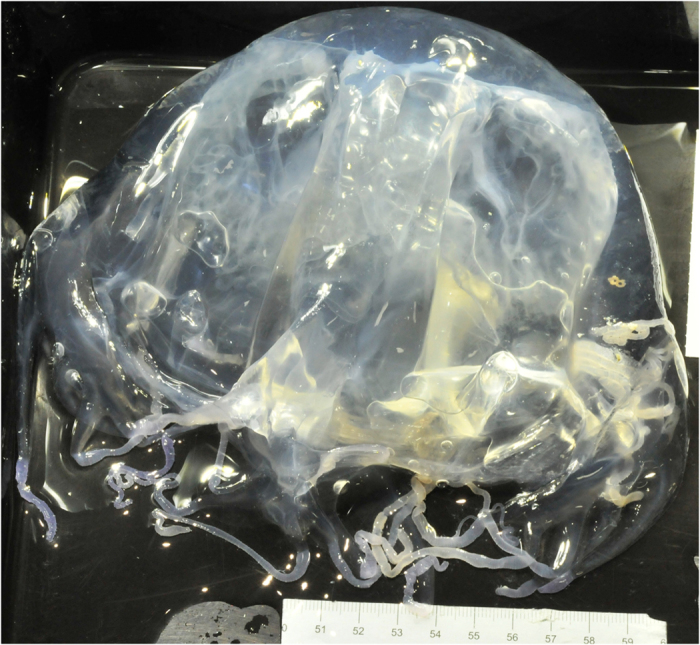
Live specimen of *Chironex* sp. captured near Degerando Island on 16 March 2015. Western Australian Museum catalogue number of this specimen is Z68783. Scale bar shows size in centimetres.

**Figure 2:**
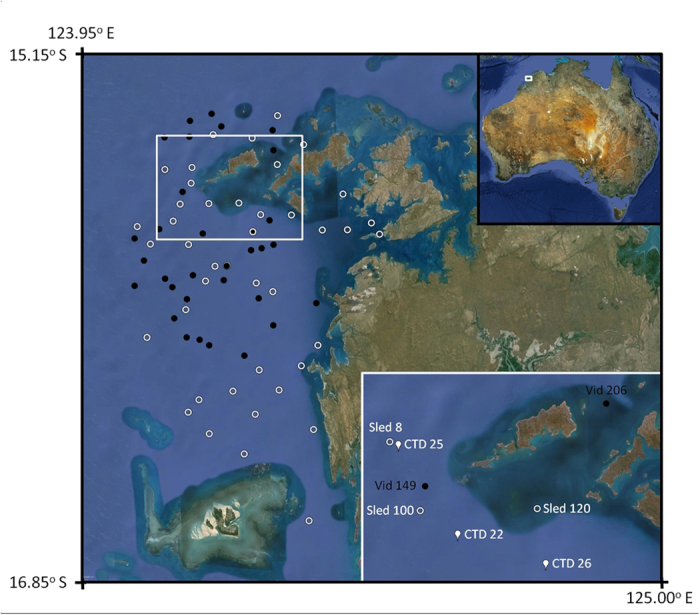
Location of sleds (white circles), tow video (white dots) in study area. The total survey area is about 2000 km^2^.Top inset shows locality of Camden Sound in north-western Australia. Lower inset shows sled, video and CTD stations (white tears) referred to in the text and in Table 1. Degerando Island is the large island located between Vid 149 and Vid 206. Map is drawn from Landsat imagery courtesy of the United States Geological Survey (www.usgs.gov).

Box jellyfish observed and captured in this study could not positively be identified as *Chironex fleckeri*. There is reported to be a species of *Chironex* from the Kimberley region of Australia that remains undescribed^10^. Specimens from this study could not be confidently determined to be *Chironex fleckeri* and have been lodged with the Western Australian Museum, registration numbers, WAM Z68783 (specimen pictured in Fig. 1, bell cube width and height when live = 12 cm) and WAM Z68784 (4 specimens). We recorded box jellyfish at just five of the 74 sites sampled across Camden Sound in an area of approximately 2000 km^2^ (Fig. 2). Only two video transects (6.1%) had jellyfish; station 149 (42 m deep) and station 206 (56 m deep) (Table 1). At tow video station 149 (Fig. 2), there were 64 box jellyfish observed along the 1500 m video transect or 0.04 jellyfish per linear m or 0.05 m^−2^. The jellyfish were not clumped; the 64 observed were distributed along the entire video tow with between zero and four (mean 1.64, standard deviation 1.36) in each one minute segment of the 40 minute video and eight of the 64 were observed as four pairs (counted in same frame). Box jellyfish were caught in just three sleds (6.8%), at station 8 (48 m deep), Station 100 (39 m) and Station 120 (53 m), catching 4, 7 and 1 individuals respectively (Table 1) and corresponding to densities of 0.02–0.14 per linear meter towed, 0.01–0.09 per m^2^ of seabed sampled by the sled and 0.04–0.28 per m^3^ of water “filtered” by the sled.

**Figure 3:**
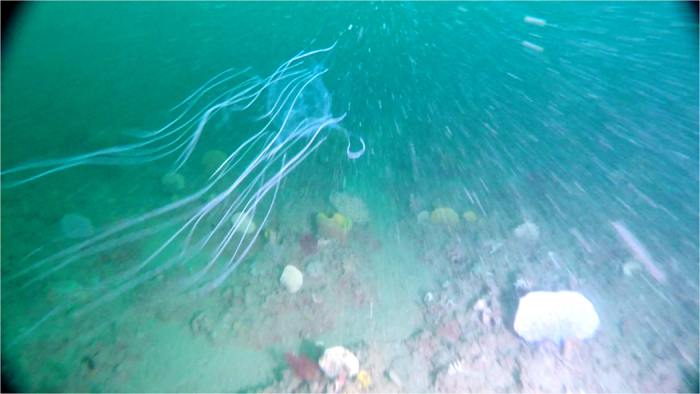
Box jellyfish close to seabed in sponge dominated benthic habitat at a depth of 42 m from Tow Video site 149 near Degerando Island in Camden Sound, north-western Australia.

Camden Sound comprises a mix of benthic habitats. At twenty three towed video stations (70%) we recorded low diversity, soft sediment, usually mud habitats. At the other ten towed video transects (30%) we recorded diverse filter feeder (sponges, ascidians, bryozoans and gorgonian) communities. Both video transects on which the box jelly fish occurred and all three of the sled stations which caught samples were also in diverse filter feeder habitats (see Fig. 3). In November 2014 we had undertaken towed video transects at the three sled sites in which we caught box jellyfish in March 2015, and there were no box jellyfish observed suggesting the phenomenon is seasonal or episodic. The water column was well mixed at all sites with temperature ranging from 30.0–31.2 ^o^C, salinity from 33.6 to 34.2 practical salinity units (PSU) and 6.14 to 6.25 mg Oxygen per litre (see Table 1).

## Discussion

The habit, habitat and early life history of *Chironex fleckeri*^6^,^7^,^8^ suggest this species is a coastal inhabitant with alternating life stages consisting of sessile polyps and small (ca. 2 mm) medusae in creek systems and a sexually mature highly mobile adult form in near-shore marine waters at depths of no more than 5 m. Our observations were also made in coastal waters among an island archipelago (jellyfish captured 6.5–8.9 km from nearest land; Degerando Island, 15.3360^o^ S, 124.196^o^ E), but from deeper water (39–56 m), 28 km offshore from the nearest mainland and creek system. The significance of the observations of *Chironex* right at the benthos on a diverse filter feeder dominated community low to medium relief reefs and the importance of this type of habitat to them remains unclear. We did not capture or observe any *Chironex* specimens on soft sediment habitats which are structurally less complex and less biodiverse, despite these habitats making up 49% of all 74 sites sampled (70% of videos and 32% of sleds). Hartwick^6^ made the point that the large medusa and long tentacles of *Chironex* made highly complex three dimensional habitats such as mangrove creeks unsuitable. The same could be said for filter feeder reefs where we made our observations. Unlike most jellyfish, box jellyfish are strong swimmers^11^, which is an advantage in highly dynamic tidal environments such as in our study site within the central Kimberley region of Western Australia where there are large tidal ranges of more than12 m and strong tidal currents^12^. To ingest prey from their tentacles into the bell, *Chironex fleckeri* usually cease swimming and orient the dorsal section of the bell downwards^7^,^13^, which would be problematic given the strong tidal currents and dense filter feeder habitat, even on low relief reef substrate. Alderslade^14^ reports that *Chironex fleckeri* feeds amongst mangroves from November to March in Darwin (Northern Territory), but moves out of the trees as soon as the slack water changes to tidal flow. This behaviour is likely to be to avoid entanglement. Another chirodropid, *Chirodectes maculatus*^15^,^16^, known only from a single specimen on the Great Barrier Reef, was also collected from shallow water above reef habitat, but it was speculated this may have been due to relocation during a storm^15^.

*Chironex fleckeri* prey predominantly on prawns^7^ but very large medusa will capture fish as well^17^. Prey items take about 5 hours to digest during which time they are visible in the medusa^7^. Most of the *Chironex* we caught in sleds or observed on the video were between 0800 h and 0930 h and none had obvious prey items being digested. A possible explanation for our observations is that the *Chironex* seek shelter from strong currents on reefs by day, where they could also hunt fish, and hunt for nocturnally emergent prawns on adjacent soft sediment habitats at night. One conflict for this explanation is that our survey was timed to coincide with neap tides and the video tow 149 on which we observed most jellyfish was during the slackest part of the tide on that day. Nevertheless currents in this area are strong at all times, but it is possible that our observation of large numbers of jellyfish on these reefs at these depths might also be explained by it being at the period of lowest tidal current. This period of lowest water movement may represent a favourable time for active hunting. Some carybdeid jellyfish are known to hunt near the seabed by swimming oblique transects along the edges of vegetative patches, where the light-dark contrast is strongest^18^. More detailed information on the behaviour of box jellyfish on these deep coastal reefs is necessary to determine whether they also pose a risk to humans in coastal areas and whether such threats can be predicted^19^. Our observations of *Chironex* in March 2015 but not November 2014 suggest their occurrence there is seasonal or episodic and is consistent with the known seasonality of the peak abundance of large medusa in March and April^10^. Although no fatalities from *Chironex* are known from the west coast of the Australian continent, there have been records of human envenomations^10^. This is the first study of *Chironex* in this region and more ecological research on this species is needed. In addition resolving the taxonomy and distribution of this species is important; given some studies attribute the *Chironex* in north-western Australia as being *C. fleckeri*^20^. Specimens from this study have been lodged with the Western Australian Museum, but more will be required to resolve the taxonomic uncertainties referred to in the literature^10^,^20^.

## Materials and Methods

The study area was in Camden Sound located in the central Kimberley region of Western Australia (Fig. 2) where a new marine park had recently been established, largely around an important humpback whale calving habitat, but where very little inventory of the benthic marine fauna had been undertaken. Thirty-three video transects and forty-one epibenthic sled stations were sampled in day light hours between 14 and 28 March 2015 from the Australian Institute of Marine Science (AIMS) research vessel RV Solander. The tow video system consisted of a tow body supporting a forward facing Watec WAT-250D digital CCD colour video camera (Watec Co. Ltd, Yamagata, Japan) with a 2.8 mm lens in a stainless steel housing and floodlight enabling on deck real time viewing^21^. The towed body also carried a downward facing Ricoh GR IV 10 megapixel digital still camera (The Ricoh Co. Ltd, Tokyo, Japan) taking pictures at 10 s intervals and a forward facing GoPro Hero 3+ video camera (GoPro Inc., San Mateo, USA). Video tows were conducted at 1.5 knots with the camera maintained approximately 50 cm above the seabed and were approximately 1500 m in length. Tow video stations were between 13.2 m and 62.3 m deep. The two stations where jellyfish were observed were 149 (16 March, 0830 h) and 206 (18 March, 1500 h) (Fig. 2). The fine-scale up and down movement of the camera is controlled by an operator who watches the video in real time and regulates the height above the seabed via an electric winch. Substrate and benthic community assemblage types were recorded in real time as was the presence of box jellyfish. For sites where box jellyfish were recorded in real time viewing, the GoPro video footage was viewed post voyage at half normal speed to accurately count their number. Jellyfish were counted if they were visible as they went past the camera port which has a known width of view at that point (84 cm). It appeared that jellyfish observed in the video stations were the same *Chironex* species as we captured at the benthic sled stations nearby.

An epibenthic sled towed along the seabed was used to sample marine benthic invertebrates and algae. The sled used had a heavy steel construction with a mouth 150 cm in width and 33 cm in height, and a cod end made from nylon mesh measuring 25 mm between knots (mesh size)^22^. Each sled was towed for 50 m, where hard bottom diverse filter feeder communities were expected, or 100 m where low diversity soft sediment habitats were expected. The exact length of each sled and video tow was recorded on the ships event logger which is linked to its global positioning system. Plants and animals were sorted and weighed on board, however only the data on cubozoans is presented here. Specimens of the box jellyfish were fixed in 100% ethanol or 4% formalin for later anatomical examination and identification. A total of 41 sled sites were undertaken at depths between 15.2 m and 61.7 m. The sled stations in which *Chironex* sp were captured were 8 (16 March, 0800 h), 100 (16 March, 0930 h) and 120 (15 March 1500 h). The density of jellyfish was calculated in four ways; per linear m of video tow, per m^2^ of video tow (using 0.84 m camera port view width × tow length), per m^2^ of seabed sampled by the sled and per m^3^ of water sampled by the sled (sled mouth area 0.495 m^2^ × tow length). A retrospective viewing was also made of videos taken in November 2014 along the same track as the three epibenthic sleds which caught box jellyfish in March 2015. Depth profiles of temperature (^o^C), salinity (PSU) and oxygen (mg/litre) were made with a Sea-BirdTM SBE19+ CTD profiler (Sea-Bird Electronics Inc., USA) at three stations in the area to determine the extent of any stratification of the water column (Table 1).

## Additional Information

**How to cite this article**: Keesing, J. K. *et al*. Abundant box jellyfish, *Chironex* sp. (Cnidaria: Cubozoa: Chirodropidae), discovered at depths of over 50 m on western Australian coastal reefs. *Sci. Rep.* **6**, 22290; doi: 10.1038/srep22290 (2016).

## Figures and Tables

**Table 1:** Summary of sled trawl catches and video observations of *Chironex* sp. and results of CTD casts in the study region.

Site /depth (m)/ Method	Medusae caught/ observed	Temperature (°C)	Salinity (PSU)	Oxygen (mg/litre)
Site 8 (48 m) Sled trawl 15.3133363^o^ S, 124.111991^o^ E	4			
Site 22 (34 m) CTD 15.4042112^o^ S, 124.173745^o^ E		30.2–31.0	33.6–34.2	6.15–6.25
Site 25 (37 m) CTD 15.322735^o^ S, 124.120257^o^ E		30.0–30.6	33.9–34.2	6.19–6.24
Site 26 (18 m) CTD 15.432224^o^ S, 124.255031^o^ E		30.9–31.2	33.7–34.0	6.14–6.17
Site 100 (39 m) Sled trawl 15.3766619^o^ S, 124.139871^o^ E	7			
Site 120 (53 m) Sled trawl 15.373057^o^ S, 124.248179^o^ E	1			
Site 149 (42 m) Tow Video 15.3542532^o^ S, 124.144693^o^ E	64			
Site 206 (56 m) Tow Video 15.2834662^o^ S, 124.314466^o^ E	2			

## References

[b1] Currie B. Clinical implications of research on the box-jellyfish *Chironex fleckeri*. Toxicon 32, 1305–1313 (1994). 7886690 10.1016/0041-0101(94)90403-0

[b2] Gershwin L. Box Jellyfish and Irukandji deaths in Australia. Australian Marine Stinger Advisory Services. http://www.stingeradvisor.com/boxydeaths.htm (2014).

[b3] Southcott R. V. Studies on Australian Cubomedusae, including a new genus and species apparently harmful to man. Aust. J. Mar. Freshwat. Res. 7, 254–280. (1956)

[b4] Lewis C. & Bentlage B. Clarifying the identity of the Japanese Habu-kurage, *Chironex yamaguchii*, sp. nov. (Cnidaria: Cubozoa: Chirodropida). Zootaxa 2030, 59–65 (2009).

[b5] Kingsford M. J. & Mooney C. J. The ecology of box jellyfishes (Cubozoa). In: Pitt K. A. & Lucas C. H., editors. Jellyfish blooms. Netherlands: Springer Publishers. pp. 267–302 (2014).

[b6] Hartwick R. F. Distribution ecology and behaviour of the early life stages of the box-jellyfish *Chironex fleckeri*. Hydrobiologia 216, 181–188 (1991).

[b7] Hamner W. M., Jones M. S. & Hamner P. P. Swimming, feeding, circulation, and vision in the Australian box jellyfish, *Chironex fleckeri* (Cnidaria: Cubozoa). Mar. Freshwat. Res. 46, 985–990 (1995).

[b8] Kingsford M. J., Seymour J. E. & O’Callaghan M. D. Abundance patterns of cubozoans on and near the Great Barrier Reef. Hydrobiologia 690, 257–268 (2012).

[b9] Lewis C. . Redescription of *Alatina alata* (Reynaud, 1830)(Cnidaria: Cubozoa) from Bonaire, Dutch Caribbean. Zootaxa. 3737(**4**), 473–487 (2013). 25112765 10.11646/zootaxa.3737.4.8 PMC4900819

[b10] Marsh L. M. & Slack-Smith S. M. Field guide to the sea stingers and other venomous and poisonous marine invertebrates of Western Australia. Perth, Western Australian Museum, 245 pp. (2010).

[b11] Gordon M. R. & Seymour J. E. Quantifying movement of the tropical Australian cubozoan *Chironex fleckeri* using acoustic telemetry. Hydrobiologia 616, 87–97 (2009).

[b12] Short A. D. Kimberley beach and barrier systems: An overview. J. Roy. Soc. West. Aust. 94, 121–132 (2011).

[b13] Barnes N. Personal communication, June 2015. Nick Barnes has made detailed observations on the behaviour of *Chironex fleckeri* over many years in his role collecting the toxin used to manufacture antivenom by the Australian Commonwealth Serum Laboratories.

[b14] Alderslade P. Personal communication, June 2015. Phil Alderslade is an expert on cnidarian biology and taxonomy. As a Curator at the Northern Territory Museum and Art Gallery (Australia) he made detailed observations of behaviour of *Chironex fleckeri* in Darwin harbour.

[b15] Cornelius P. F. S., Fenner P. J. & Hore R. R. *Chiropsalmus maculatus* sp. nov., a cubomedusa from the Great Barrier Reef. Memoirs of the Queensland Museum 51(2), 399–405 (2013).

[b16] Gershwin L. Comments on *Chiropsalmus* (Cnidaria: Cubozoa: Chirodropida): a preliminary revision of the Chiropsalmidae, with descriptions of two new species. Zootaxa 1231, 1–42 (2006).

[b17] Carrette T., Alderslade P. & Seymour J. Nematocyst ratio and prey in two Australian cubomedusans, *Chironex fleckeri* and *Chiropsalmus* sp. Toxicon 40, 1547–1551 (2002). 12419505 10.1016/s0041-0101(02)00168-x

[b18] Matsumoto G. I. Observations on the anatomy and behaviour of the cubozoan *Carybdea rastonii* Haacke. Mar. Freshwat. Behav. Physiol. 26, 139–148 (1995).

[b19] Gershwin L., Condie S. A., Mansbridge J. V. & Richardson A. J. Dangerous jellyfish blooms are predictable. J. Roy. Soc. Interface 11(96), 20131168, doi: 10.1098/rsif.2013.1168. (2014). 24829278 PMC4032527

[b20] Bentlage B., Peterson A. T. & Cartwright P. Inferring distributions of chirodropid box-jellyfishes (Cnidaria: Cubozoa) in geographic and ecological space using ecological niche modeling. Mar. Ecol. Prog. Ser. 384, 121–33 (2009).

[b21] Colquhoun J. . Ningaloo Reef Marine Park Deepwater Benthic Biodiversity Survey. Report for Western Australian Marine Science Institution (WAMSI). Australian Institute of Marine Science, Perth, p. 143. (2007).

[b22] Keesing J. K. . Marine benthic flora and fauna of Gourdon Bay and the Dampier Peninsula in the Kimberley region of north-western Australia. J. Royal. Soc. West. Aust. 94, 285–301 (2011).

